# Climate Change and Public Health Policy: Translating the Science 

**DOI:** 10.3390/ijerph110100013

**Published:** 2013-12-19

**Authors:** Marieta Braks, Rijk van Ginkel, William Wint, Luigi Sedda, Hein Sprong

**Affiliations:** 1 Centre for Zoonoses and Environmental Microbiology, National Institute for Public Health and the Environment, P.O. Box 1, Bilthoven 3720 BA, The Netherlands; E-Mail: hein.sprong@rivm.nl; 2 Department of Infectious Disease Control, Municipal Public Health Service Rotterdam-Rijnmond, P.O. Box 70032, Rotterdam 3000 LP, The Netherlands; E-Mail: r.vanginkel@rotterdam.nl; 3 Environmental Research Group Oxford, Department of Zoology, University of Oxford, South Parks Road, Oxford OX1 3PS, UK; E-Mail: william.wint@gmail.com; 4 Department of Zoology, University of Oxford, South Parks Road, Oxford OX1 3PS, UK; E-Mail: luigi.sedda@zoo.ox.ac.uk

**Keywords:** climate change, public health, scientific evidence, pitfalls, mosquito borne diseases

## Abstract

Public health authorities are required to prepare for future threats and need predictions of the likely impact of climate change on public health risks. They may get overwhelmed by the volume of heterogeneous information in scientific articles and risk relying purely on the public opinion articles which focus mainly on global warming trends, and leave out many other relevant factors. In the current paper, we discuss various scientific approaches investigating climate change and its possible impact on public health and discuss their different roles and functions in unraveling the complexity of the subject. It is not our objective to review the available literature or to make predictions for certain diseases or countries, but rather to evaluate the applicability of scientific research articles on climate change to evidence-based public health decisions. In the context of mosquito borne diseases, we identify common pitfalls to watch out for when assessing scientific research on the impact of climate change on human health. We aim to provide guidance through the plethora of scientific papers and views on the impact of climate change on human health to those new to the subject, as well as to remind public health experts of its multifactorial and multidisciplinary character.

## 1. Introduction

The females of most mosquitoes need to feed on the blood of living vertebrates including humans, to successfully reproduce, and in the process may transmit pathogens (viruses, bacteria or parasites) and so serve as vectors of these diseases. Mosquito-borne diseases are especially important vector-borne diseases with malaria, dengue and yellow fever alone affecting millions of people every year (Table 1).

**Table 1:** Public health related characteristics of important mosquito borne diseases.

Disease	Annual Global Cases ^1^	Pathogen	Vector Genus	Infectious Period	Prophylaxis	Vaccine	Curative Medicine
Malaria	451 million ^2^	*Plasmodium*	*Anopheles*	Up to year ^7^	√	-	√
Dengue	96 million ^3^	Flavivirus	*Aedes*	3–5 days	-	-	-
Yellow fever	200,000 ^ 4^	Flavivirus	*Aedes*	3–5 days	-	√	-
Japanese Encephalitis	67,900 ^5^	Flavivirus	*Culex*	dead end host	-	√	-
West Nile fever	20, 000 ^6^	Flavivirus	*Culex*	dead end host	-	- ^9^	-
Chikungunya	Epidemic	Alphavirus	*Aedes*	6–7 days	-	-	-
Rift Valley fever	Epidemic	Phlebovirus	*Culex/Aedes*	short ^8^	-	-	-

Note: ^1^ Case estimates, exact numbers not available; ^2^ [1,2]; ^3^ [3] Clinical cases only, ¾ of dengue infections are apparent; ^4^ Who Factsheet N° 100 May 2013; ^5^ [4]; ^6^ [5]; ^7^ Depending on *Plasmodium* species but when untreated up to a year, exception of *Plasmodium vivax* with prolonged incubation period up to 5 years; ^8^ [6] Humans theoretical reservoir (low epidemiological significance); ^9^ Veterinary vaccines available for horses.

Worldwide, the most important mosquito vector species are members of three genera, *Aedes*, *Culex* and *Anopheles*, each having its own set of climatic and environmental drivers and constraints. Not only can a species occur within its natural geographical range (past or present) and dispersal potential (indigenous species), but it can also occur outside this range through various introduction routes (exotic species). An exotic (or invasive) species may subsequently establish and spread causing economic or environmental impact or harm to human health [7]. The yellow fever mosquito, *Aedes aegypti*, for example is indigenous to Africa, but is an exotic species in The Netherlands where it has been introduced, but cannot establish due to prevailing climatic conditions [8], and an invasive mosquito in Madeira where it has been established since 2002, and was a vector for a dengue epidemic in 2012 [9]. 

**Figure 1:**
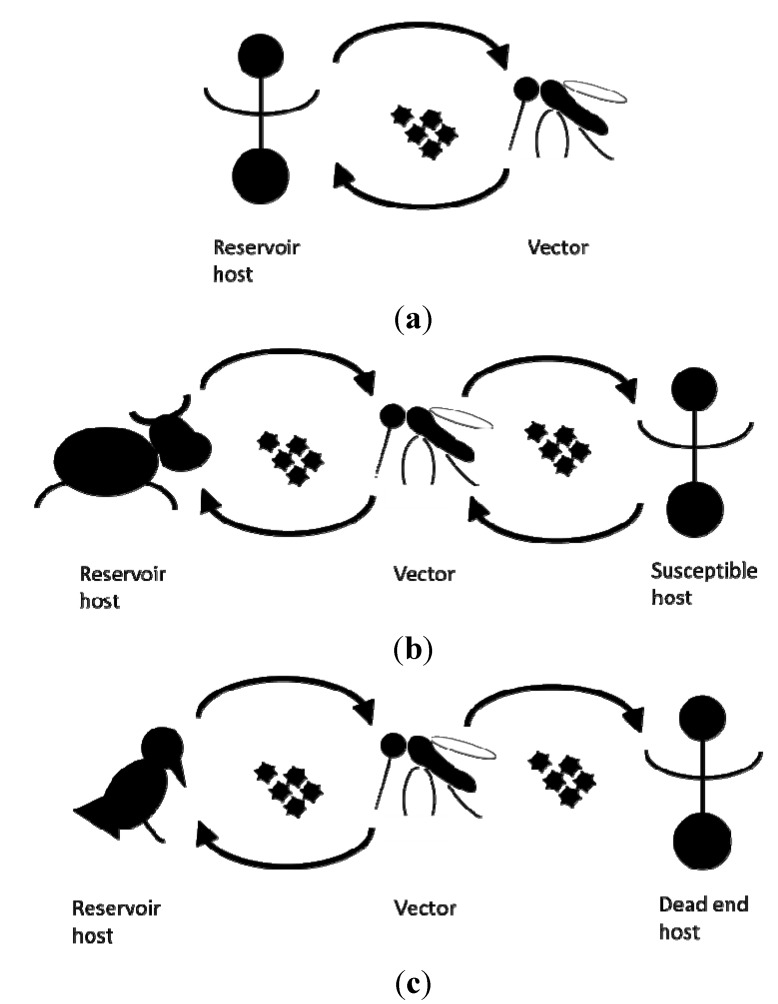
Schematic representation of the transmission cycles of (**a**) dengue virus, (**b**) Rift valley fever virus, (**c**) West Nile fever virus.

An established vector population alone does not pose an immediate risk without another critical element: the presence of the pathogen itself. Depending on the pathogen, an infection can cause disease in human, livestock and wildlife. Some mosquito borne pathogens are maintained in a human-vector-human cycle, whilst the lifecycles of others also involve (wild) reservoir host animals. Here, humans frequently act as dead end hosts from which pathogens are not transmitted to other susceptible hosts [10] (Figure 1). 

Whether actual transmission of mosquito borne pathogens can occur in a specific time and place depends on the vector capacity, a parameter combining the level of intrinsic (genetic and physiological) ability of the mosquito species present to transmit the pathogen (vector competence) with the other factors affecting transmission such as mosquito population and host reservoir density, host preferences, and biting rates [11]. As long ago as 1966, Pavloskiy proposed the concept of focality or nidality of diseases, in which pathogens are associated with specific landscape. The dimensions of possible transmission thus largely depend on the vector bionomics and pathogen natural history [12], including its vulnerable primary hosts, either humans or other vertebrates. 

Climate changes may affect both these dimensions, and therefore the spatio-temporal distribution of possible transmission. Using scientific methods, knowledge of these complex systems needs to be accumulated and organized in the form of testable explanations and predictions to support public health policymakers in making decisions on the way forward. However, the nature of scientific information, which is often extensive, complex, uncertain and ambiguous, also complicates the development of evidence-based health policies [13,14] by decision makers who may not be fully trained in the disciplines needed to evaluate the evidence.

In the following sections, we discuss the advantages, disadvantages, pitfalls and lessons, of the different scientific approaches for the development of public health strategies to prepare for climate change. We examine four topics, namely: global warming *versus* global change, models *versus* the real world, retrospective *versus* prospective studies, and generalized *versus* contextual approaches. We identify a number of lessons to be learned and by doing so hope to support public health policymakers in making decisions their future strategies.

## 2. Pitfalls and Lessons

### 2.1. Global Warming versus Global Change

Mosquito vectors, like all cold-blooded animals, are obviously sensitive to (changes in) temperature and, provided the temperature does not exceed a lethal threshold, rising temperatures usually mean more rapid development of the mosquito and replication rate of the pathogen in the mosquito or extrinsic incubation period. Consequently, the majority of climate change research has focused on the assessments of the effect of increasing temperatures on pathogen transmission through the modulation of life history traits of the vector [15]. However, this global warming is telling only part of the story of climate change. Climate change also entails changes in rainfall and wind patterns and consequently relative humidity, rising sea levels and increasing UV radiation [16,17,18]. Consequently, climate change impacts land use and land cover, crop suitability and agricultural patterns and human behavior. The spatial and temporal heterogeneity of climate change may generate novel climates and environments in many geographic regions [19]. Due to their dependence for reproduction on water bodies, mosquitoes (and the diseases they transmit) are particularly sensitive to changes in quantity and quality of these aquatic breeding sites due to for example increased precipitation or drought [20]. Populations of hosts, competitors and natural enemies of vectors are also affected [18,21]. While the outer limits of a species distribution are largely determined by climatic or environmental factors, biotic interactions have also been shown to play an important role in shaping populations within those extents [22]. Dispersal, via human facilitated invasion, is an additional factor; even if conditions are ideal species may not occur, simply because they have not reached the place [23].

A number of adaptations to the effects of climate change can be anticipated. The introduction of green (vegetation) and blue (water) infrastructure in cities to alleviate urban heat islands [24], and construction of water retention and storage facilities to mitigate the impact of changing precipitation intensities and frequencies [25] are examples of adaptation on a community level possibly likely affecting urban mosquito populations. On a more individual level, people might either spend more time outdoors in the country side or in air conditioned locations, thereby affecting their possible exposure to mosquito bites and potentially to pathogens [26]. 

Undoubtedly, both the incidence and geographical distribution of vector borne diseases are expected to change as a general result of direct and indirect climate change [22]. However, global changes in land use, trade and travel patterns, leisure time, urbanisation, and standard of living play an important role in the distribution of vectors, reservoirs and pathogens, and consequently in the emergence of vector borne diseases [12,27,28,29,30,31,32]. The vulnerability to outbreaks differs between human populations [33,34]. Whether a mosquito borne disease will actually emerge in a suitable particular place at a particular time, will also largely depend on the array of interventions that can be applied to interrupt disease transmission or reduce disease burden, by personal protection, vaccines or curative medicine (Table 1), or vector management. While vaccines or curative medicine, when available, may prevent or restrict the disease burden in people, zoonotic (within animal hosts) pathogen transmission is often not stopped. 

Lesson 1: Over-emphasizing the importance of climate in disease emergence is misleading [33]. Climate change may affect disease burden directly and indirectly in many ways, but needs to be considered alongside a number of other factors, which is a complex process. 

### 2.2. Models versus Real World

Predicting the impact of climate change on public health in general and mosquito borne diseases in particular is challenging. In part this is due to the uncertainty in predicting the multifactorial local effects of global changes in climate [35]. But even when assuming a certain scenario as a fact, huge uncertainties about its effect on health remain. To comprehend the complex relationships between climate change and mosquito borne diseases they have been broken down into components. Data on the vector bionomics and pathogen kinetics are predominantly acquired using basic biological observational and experimental research. The latter studies are invaluable for examining the validity of hypotheses under controlled conditions. The validity of laboratory data in the outside world is questionable as responses to varying conditions or key parameters can be missed. Recent studies, however, are increasingly considering the impacts of the changing environment on mosquito bionomics [36,37].

To understand complex systems, to study the effects of different components, and to make predictions about their behaviour, mathematical modelling techniques are used. These models can be broadly divided into two categories: mechanistic and statistical. Reiner *et al*. defined mechanistic models as those in which the equations, formulae or computer simulations are based on assumptions about the processes or proximate causal mechanisms under consideration [38]. In the course of developing a mechanistic model, the various steps in disease transmission are described. For lack of other data, laboratory results on, for example, critical thresholds and development rates form the input data for process based predictions on distributions of vectors and diseases. A widely used measure of the probability of establishment of a vector borne disease is the basic reproduction number, also referred to as R0 [31]. The value of R0 depends, among other factors, on parameters such as the rate of development of the pathogen, the number of times the vector bites the hosts, the survival rate of the vectors and the population abundance and seasonality of both vectors and hosts. Analogous to laboratory studies, mechanistic models examine the validity of hypotheses under controlled mathematical conditions. These models are developed with specific aims outlined in a certain context and with an underlying set of rules and assumptions. Knowledge of the context and limitations of the models is essential when interpreting the results. Unfortunately, conclusions are often drawn outside the validity range of the assumptions—by the researcher themselves in some cases—but more often by the reader.

Statistical models are commonly used to identify constraints and drivers, including climate, that are currently associated with a vector and/or disease distribution or spread but without identifying the underlying process [39]. Roger *et al*. [22], states “Many (species) distribution modelling approaches involve a sort of data mining to match pattern of points in a database to sets of environmental and other predictors. It is truism that any pattern can be matched as long as sufficient variables, thresholds and break points are allowed in the models”. The fact that underlying processes are not identified hampers the design of intervention measures based on the model results. They are, however, the only technique available if, as is often the case, sufficient details of transmission dynamics are not available, and they do provide estimates of their accuracy. Successful outbreak predictions have been made using this approach [40], [41]. Note that such models need to be evaluated very carefully as it is often not clear how the model outputs actually relate to real disease risk: as pointed out earlier the presence of a vector does not guarantee a disease will occur, nor does the presence of a disease always mean it will persist or spread. 

Future threats of vector borne diseases can also be assessed combining both modelling approaches [42,43]. Among others, Hartemink [42] demonstrated that the risk of emergence of vector borne zoonoses displays high spatial and temporal variation due to interplay of multiple factors, using this integrated method. Uncertainty and sensitivity analyses are used to investigate the accuracy and robustness of a study when the study includes some form of model-based and/or stochastic approach. Another approach is to assume certain rather simple constraints on a species performance, without specifying in advance where are the most important variables [22]. 

Most of current models belong to the reductive analysis approach, aiming to describe patterns and understand how various processes interplay. The output of the model largely depends on the scope (minimise the error or maximise the information), assumptions and the choice of the input data [22,44]. The fact that different models produce different outputs is obviously challenging for developing evidence-based policies.

Lesson 2: Understanding the conditions and assumptions that underlie both laboratory and modelled data are essential when interpreting the outcome; extrapolation to the real world often lies outside the validity range of the research.

### 2.3. Retrospective versus Prospective Studies

An important classifier of investigations into the relation between climate change and vector borne diseases is whether a study looks back (retrospective) or forward (prospective) in time. In the former, explanatory variables from the past are analysed to explain the current situation, events or processes, whilst in the latter, these explanatory factors drivers and constraints, (which themselves may be projected) are used to predict the disease in the future. 

Retrospective studies have the advantage that factors are examined in relation to an outcome that is established at the start of the study, when the process is stabilised or in equilibrium, and always statistically bounded [45]. Retrospective researchers, however, have to be alert to potential sources of bias, changes in relationships according to the predictor levels (non-linearity), and the presence of confounding or proxy variables. Bias is a systematic error that leads to an incorrect estimate of effect or association. The non-linearity of the covariates means that the relationship between the outcome and the variable could change according to the level of the variable, so that if we predict the outcome at values out of the variable range used for the retrospective study the analysis is statistically invalid or at least affected by ignorance (that is a component of uncertainty). A confounding or proxy variable is one that, for example, varies in the same way as the real cause of a change in a disease, but is not actually the cause. Indeed, climate may be a confounding variable for any increase of mosquito borne disease incidence or outbreak that has occurred during recent decades (see Box). Establishing actual causality is often overlooked in the popular debate contributing to the general perception that climate change affects vector borne disease emergence. Beware that rare events with major impact, such as a disease outbreak, are frequently rationalized by hindsight, as if it could have been expected [45]. 

Prospective studies make use of the important drivers and constraints, climatic or not, identified in retrospective studies and then utilize them to estimate risk of occurrence in the future. In such risk assessment, the likelihood that a specified negative event will occur is determined [46]. It indicates the presence of preconditions for an outbreak, but it does not tell you whether it actually will occur, and may not specify its timing, size, location and spatial spread. The latter is still not well understood or appreciated by public health experts, which results in criticism if outbreaks happen in areas of low likelihood or nothing happens in areas with high likelihood. Moreover, if a prediction of elevated risk triggers effective timely and preventive intervention, the outbreak does not happen and the public wonders why the resources were expended to control something that did not occur. On the other hand, science may have provided answers to questions not asked by public health experts. While academics produce maps with spatial distribution of accurate risk outputs of mathematical modelling, the public health experts may want a simple description of the risk: present or absent, or, if there is a risk, they need to know the best and worst case scenarios rather than a prediction of the most likely risk levels. There is a difference, of course, between predicting an increase in an old or endemic problem and the emergence of a new problem. The latter is inherently more uncertain.

Lesson 3: There is a fundamental difference between knowing the past and predicting the future. 

Lesson 4: High impact rare events occur beyond the realms of normal expectations. 

Lesson 5: Researchers may not appreciate what information the Public Health professionals actually need to make appropriate decisions, and better communication between the two groups is badly needed.

### 2.4. Generalized versus Contextual Approach

From the preceding discussions it is clear that making generalized statements beyond “climate change is a driver for mosquito borne diseases” is misguided. Even that simple statement only holds true when it embraces both agonistic and antagonistic drivers that favour or hinder vector borne diseases, respectively. The spatial as well as temporal variation in the occurrence of a certain mosquito borne disease is linked to geographic differences in its constraints. Whilst in the tropics, conditions might change beyond the tolerance levels of any given mosquito species, this is not expected to occur in temperate Europe and it is assumed that rising temperature will consistently speed up mosquito vector development and the pathogens in it [18]. Changes in relative humidity in temperate zones may have minimal effects on the adult population of species that predominately inhabit wetlands, as humid shelters should remain relatively abundant. However, adult mosquitoes, inhabiting urban areas by breeding in artificial containers, are likely to be affected negatively by a decreasing relative humidity as a result of the development of urban heat islands [16]. Besides changes in precipitation, rising sea levels will affect the availability and suitability of mosquito breeding sites. Saline and brackish water bodies in coastal areas will increase [47], probably at the expense of fresh water bodies and their aquatic inhabitants. Such changes will, however, create more breeding sites for salinophilic species breeding such as the Dutch malaria mosquito *An. atroparvus*. Rising sea levels could also potentially reverse the historical reduction in the habitat of this species that occurred in the 1900s [48]. While only examples of the effect of climate change on the vector were given, the same principles hold for hosts, pathogens and with that for mosquito borne diseases. 

Already included in the word, climate change refers to variables changing relative to the norm and not to absolute values. An outbreak is also, by definition, an anomaly in expected cases per year. Because of alleviation of the prevailing constraints, outbreaks are noticed in places where they normally do not occur. Finding the causes of mosquito borne disease emergence dominates the research into climate change and vector borne diseases, effectively ignoring the fact that on many occasions, diseases did not emerge on other occasions when conditions were apparently similar, a pitfall of retrospective studies as mentioned earlier. Where and when it happens depends on whether the limiting factor(s) was removed by climate, environmental, socio-economic or other change. Cataloging all possible evidence of a past or predicted impact on any mosquito borne disease, sometime, somewhere without putting it in perspective does not bring public health authorities closer to knowing what to do to be prepared for the in the future. Many such reviews nevertheless exist [20,28,49,50,51,52,53,54]. There is a need for an approach that brings us beyond the recognition and appreciation of the complexity of climate change and public health, and provides contextual guidance. 

Lesson 6: A contextual approach is needed to understand climate and human health and to develop public health strategies. 

## 3. The Way Forward

Public health authorities are required to prepare for future threats and need predictions of the likely impact of changing climate on public health risks. Usually they focus their preparations on their own geographical region. The threat level of a mosquito borne disease for a particular country can be categorized into one of five contexts, based on the presence or absence of three important facets important for public health: human cases, pathogens and vectors (Table 2) [55]. Mosquito borne diseases pose no risk when neither the pathogen nor vector is present (context 5). Here, future establishment of the vector after introduction is the main concern and information on potential impact of climate change on the disease can be ignored by the national health authority. However, if a disease is endemic in a country (context 1), climate change may affect the size of the established vector population or rate of transmission from vectors to hosts, and consequentially the incidence of human cases. In countries where an established vector population of a vector borne disease is present (context 1–3), the current climatic and environmental conditions are obviously suitable for the vector, but whether the population size will increase or decrease in response to climate change depends on the species-specific requirements. If no established vector population is present (yet) (context 4–5), the current climatic and environmental conditions may either be unsuitable or be suitable, but the vector has yet to reach the region. It is important to keep in mind that the context of a particular mosquito borne disease can differ between countries; West Nile fever belongs to context 3 in the Netherlands, but to context 1 in Italy.

**Table 2:** Current situations of mosquito borne diseases for Western Europe, here delimited by Belgium, Netherlands and UK [55,56].

Context	Locally Acquired Human Case	Pathogen	Vector	Mosquito Borne Diseases in Western Europe
1a	√ (every year)	√	√	*No examples*
1b	√ (not every year)	√	√	*No examples*
2	*-*	√	√	Heartworm [57], Usutu [58], Ockelbo [59]
3	*-*	-	√	West Nile virus [60], Malaria [61], Rift Valley Fever [62]
4	*-*	√	-	Chikungunya; Dengue [63]
5	*-*	-	-	Japanese encephalitis *

Note: * Potentially European mosquitoes are competent to transmit JEV [64], but this has not been validated.

Factors determining the success of a novel or exotic species in a new location differ between the sequential phases, namely the introduction, establishment, and geographic spread. For mosquitoes, arrival in a new area can occur through active migration or passive transport mediated by wind, or by trade and travel movements. In the last thirty years, global trade and travel has increased exponentially, resulting in an increase of the risks of the arrival of novel mosquito species [43], for example, by the trade of used tires or in airplanes [44]. Establishment and subsequent geographic spread of a species depends on whether the introduced species encounters suitable climatic and ecological conditions at the new location. The chances for this to happen, in general, are considered rather small [45], except for a few notorious invasive mosquito species such as *Ae. aegypti* and *Ae. albopictus* [65].

While the establishment and spread of a mosquito species after its introduction to a new area are transient processes, the effect of the arrival of a novel pathogen in an area with an established vector population can be very rapid and substantial, as seen with West Nile virus introduction in USA [46]. However, since introductions are often only noticed when causing a significant disease burden, no real insight exists on how often pathogens arrive but do not become established or do not cause an outbreak of disease. As with vectors, the chances of successful establishment and spread of pathogens are also considered to be rather small, considering its dependence on enabling hosts, vectors and environmental and climatic conditions. The chances on disease burden can largely differ between human populations with different socio-economic statuses [66].

For a single country, basic information on vector and host populations present and potentially circulating pathogens are required to assess the contexts of mosquito borne diseases. Subsequently, based on their context, the best surveillance strategy can be developed for each mosquito borne disease, depending on the potential prospectives for action and the costs/benefit analysis. In a time of grim governmental budget cuts, focusing on interventions that achieve the largest health gain per euro spent ever more necessary. For some mosquito borne diseases, taking action (e.g., preventing the establishment of invasive mosquitoes) even when as yet there is no disease, might be more effective than waiting until the disease appears [55]. Once a decision to intervene to decrease the disease burden (or group/category of diseases) or to mitigate a threat has been made, surveillance should be implemented in order to measure the effectiveness of the intervention [8]. 

The described contextual surveillance for vector borne disease can be easily extended with veterinary and wildlife health along with public health to be applicable in a One Health approach, Surveillance programs providing knowledge on the current distributions of the disease, the pathogen and the vector, are vital in the development of appropriate One Health policies.

## 4. Conclusions

Disease emergence in its own right is inherently complex and uncertain, let alone the impact of climate change on this. While the recognition of the complexity of climate change and disease emergence is important, public health authorities need to focus on developing and maintaining contextual surveillance programs.

Climate change, entailing increasing temperature, changes in patterns of precipitation and other meteorological factors, and rises in the number of extreme events, is expected to affect the emergence, incidence and geographical distribution of vector borne diseases. Predictions on the direction and size of these effects are needed to inform an optimal public health response. Complex transmission pathways, typical for vector borne diseases, as well as regional climate change projections are often insufficiently understood and largely uncertain, hence any combination can produce misleading results [67]. In addition, many factors other than climate have been identified as having a significant effect on whether vector borne diseases emerge or not [12,27,28,29,30,31,54]: these include the increase in urbanization, trade and travel, socio-economic and environmental changes as well as distinct differences in vulnerabilities between human populations [34,68,69,70,71,72]. Various lists of a(nta)gonistic drivers for emergence of infectious diseases, including climate change exist. While the majority of recent publications acknowledge the overwhelming complexities, unknowns and uncertainties of the relation between climate change and vector borne disease, the generalized idea that the transmission of vector borne diseases is favoured by climate change remains the most widely held working hypothesis and dominates the public debate. By identifying major pitfalls of this working hypothesis and highlighting specific lessons to be learned, we hope to support public health advisors in the development of local evidence-based public health strategies.

Box: Mosquito-borne diseases in Europe

The decades following the eradication of malaria in the 1960s and 70s, mosquito borne diseases were not considered important problems for public health in Europe. In this period, only incidental cases and infrequent outbreaks of West Nile fever had been observed except in Italy [73], and the disease burden of the other mosquito borne diseases has also been also low [74]. Endemic malaria cases only occurred in six countries from the WHO European region, (Azerbaijan, Georgia, Kyrgyzstan, Tajikistan, Turkey, and Uzbekistan).

However, in recent decades the situation with mosquito borne diseases seems to have changed. Between 1996 and 1998, serious outbreaks of West Nile virus in Romania, Russia, Italy and Israel have occurred. Since then, WNV circulation has been reported from multiple countries inside the European Union (EU) including France, Greece, Italy, Portugal, Romania, Serbia and Spain, and from close neighbours: Turkey, Russia, Morocco and Israel [73]. Further, in 2007 more than 200 people fell ill from the first European outbreak of chikungunya in Italy [75]. Subsequently, in 2010 the first autochthonous cases of chikungunya and/or dengue were detected in Southern France and Croatia and transmitted by the Asian tiger mosquito, *Ae. albopictus.* In 2012, Madeira experienced a significant dengue outbreak vectored by *Ae. aegypti* [9]. Between 2009 and 2012, Greece has also experienced several clusters of locally acquired malaria, predominantly caused by the recent steady introduction of non-symptomatic gametocyte immigrant workers infecting the local malaria mosquito population [76]. In 2009, the first known human cases of Usutu virus infections were described in Italy [77]. In 2008 in his consideration of mosquito borne viruses occurring in Europe since the 20th century, Hubalek [31,56] listed eight viruses that are proven pathogenic to humans, belonging to three families *Togaviridae* (sindbis, chikungunya), *Flaviviridae* (West Nile, dengue) and *Bunyaviridae* (Batai, Tahyna, Snowshoe hare, Inkoo). The recent reports of Usutu (*Flaviviridae*) infections in humans [77] brings that number to nine (Table 2).

Correlation of these recent events with the increasing recognition of the process of climate change may have fuelled speculations about causality and implications for the future [31]. Convincing evidence, however, exists that non-climatic processes were the main determinants of these outbreaks. Major changes in the global distribution of chikungunya, for example, have been shown in part to be due to a genetic adaptation of the virus. While its principle vector used to be the yellow fever mosquito, a recent mutation, has meant that it is effectively transmitted by the Asian tiger mosquito, a more temperate species [78]. This virus quickly reached Italy through a travelling viraemic patient. There it found a highly effective resident vector population and infected many people [75]. The latter also holds for the recent autochthonous cases of dengue and chikungunya in France and Croatia. The current occurrence of multiple autochthonous *vivax*- malaria in Greece is probably caused by a steady introduction of non-symptomatic gametocyte immigrant workers infecting the local malaria mosquito population [76].

Such events imply that these vector borne disease outbreaks occurred because of the arrival of a pathogen in a location suitable for transmission. The chance for such introductions has increased due to the recent enormous growth in trade and travel movements [79], which has increased the vulnerability of Western Europe [80] to introductions from abroad. Since climate change does not seem to play a major role in the introduction of these pathogens, the question arises as to whether it has (retrospective) or will (prospective) facilitate the establishment or spread of diseases. In Western Europe, temperature constraints for life history traits of mosquitoes and the pathogens they carry may be relaxed and transmission season may be extended, which may have and may in future increase the suitability of a region to support some mosquito borne disease [80]. In the light of the many changes occurring, new players may also surface in mosquito borne disease epidemiology, as illustrated by the human-induced expanded distribution of *An. plumbeus* in Belgium [61].
